# Central melanopsin projections in the diurnal rodent, *Arvicanthis niloticus*

**DOI:** 10.3389/fnana.2015.00093

**Published:** 2015-07-14

**Authors:** Jennifer L. Langel, Laura Smale, Gema Esquiva, Jens Hannibal

**Affiliations:** ^1^Neuroscience Program, Michigan State UniversityEast Lansing, MI, USA; ^2^Department of Psychology, Michigan State UniversityEast Lansing, MI, USA; ^3^Department of Zoology, Michigan State UniversityEast Lansing, MI, USA; ^4^Department of Clinical Biochemistry, Bispebjerg Hospital, University of CopenhagenCopenhagen, Denmark; ^5^Department of Physiology, Genetics and Microbiology, University of AlicanteAlicante, Spain

**Keywords:** retinal projections, PACAP, melanopsin, circadian rhythms, Nile grass rat, suprachiasmatic nucleus, LGN, pretectum

## Abstract

The direct effects of photic stimuli on behavior are very different in diurnal and nocturnal species, as light stimulates an increase in activity in the former and a decrease in the latter. Studies of nocturnal mice have implicated a select population of retinal ganglion cells that are intrinsically photosensitive (ipRGCs) in mediation of these acute responses to light. ipRGCs are photosensitive due to the expression of the photopigment melanopsin; these cells use glutamate and pituitary adenylate cyclase-activating polypeptide (PACAP) as neurotransmitters. PACAP is useful for the study of central ipRGC projections because, in the retina, it is found exclusively within melanopsin cells. Little is known about the central projections of ipRGCs in diurnal species. Here, we first characterized these cells in the retina of the diurnal Nile grass rat using immunohistochemistry (IHC). The same basic subtypes of melanopsin cells that have been described in other mammals were present, but nearly 25% of them were displaced, primarily in its superior region. PACAP was present in 87.7% of all melanopsin cells, while 97.4% of PACAP cells contained melanopsin. We then investigated central projections of ipRGCs by examining the distribution of immunoreactive PACAP fibers in intact and enucleated animals. This revealed evidence that these cells project to the suprachiasmatic nucleus, lateral geniculate nucleus (LGN), pretectum, and superior colliculus. This distribution was confirmed with injections of cholera toxin subunit β coupled with Alexa Fluor 488 in one eye and Alexa Fluor 594 in the other, combined with IHC staining of PACAP. These studies also revealed that the ventral and dorsal LGN and the caudal olivary pretectal nucleus receive less innervation from ipRGCs than that reported in nocturnal rodents. Overall, these data suggest that although ipRGCs and their projections are very similar in diurnal and nocturnal rodents, they may not be identical.

## Introduction

In mammals, light has a strong impact on daily activity rhythms by synchronizing an organism’s internal clock with rhythms in the external environment (i.e., light entrainment) and through more acute effects on general activity (a phenomenon known as masking). While entraining effects of light on the internal clock are very similar in diurnal and nocturnal species (reviewed in Smale et al., 2003), masking effects are quite different, with light increasing locomotor activity in diurnal species and decreasing activity and inducing sleep in nocturnal ones (Aschoff and Vongoetz, 1989; Redlin and Mrosovsky, 1999; Shuboni et al., 2012). The neural pathways contributing to these differences are not well understood but they are likely to involve circuits that receive signals, directly or indirectly, from a subset of retinal ganglion cells that are intrinsically photosensitive (ipRGCs). These cells, which contain the photopigment melanopsin, are important for non-image-forming visual functions, such as entrainment, masking, melatonin suppression, and regulation of the pupillary light reflex (PLR). Within the last decade it has been shown that these light regulated functions are driven from signals generated not only from melanopsin activation, but also from the classical photoreceptors, rods and cones. When genetically removing rods, cones, or melanopsin, masking still occurs (Mrosovsky et al., 2001; Panda et al., 2002), but removal of all three types of photoreceptors or ipRGCs eliminates masking and entrainment (Hattar et al., 2003; Panda et al., 2003; Goz et al., 2008; Guler et al., 2008; Hatori et al., 2008). It is clear, therefore, that ipRGCs play an essential role in transmission of photic signals that lead to a masking response in nocturnal mice. The question of whether this is also the case in diurnal species is an open one, and differences along circuits that process signals from ipRGCs could theoretically contribute the differences in the acute effects of light on activity in day- and night-active animals.

Many brain areas such as the suprachiasmatic nucleus (SCN), lateral geniculate nucleus (LGN), pretectum, and superior colliculus (SC) are innervated by ipRGCs in nocturnal mice, as indicated by studies of transgenic models (Hattar et al., 2006; Brown et al., 2010; Ecker et al., 2010). Another approach to determine central projections of ipRGCs in other animals has been to use a combination of a classical anterograde tracer (cholera toxin subunit β; CT-β) injected into the eye and co-staining for one marker of ipRGC fibers, the neuropeptide, pituitary adenylate cyclase-activating polypeptide (PACAP), in retinal target areas of the brain (Bergstrom et al., 2003; Hannibal and Fahrenkrug, 2004; Hannibal et al., 2014). In the retina of mammals examined so far, PACAP is exclusively expressed within RGCs that contain melanopsin both in diurnal and nocturnal species (Hannibal et al., 2000, 2002, 2004, 2014; Bergstrom et al., 2003).

One diurnal model available for investigation of neural pathways involved in masking is the Nile grass rat (*Arvicanthis niloticus*). These animals are native to sub-Saharan Africa and are diurnal both in the field and in the laboratory (Katona and Smale, 1997; McElhinny et al., 1997; Blanchong and Smale, 2000). They have a cone-rich retina, as do most other diurnal species; specifically, the ratio of cones to rods is 10 times higher in the Nile grass rat than in typical nocturnal rodents (Gaillard et al., 2008; Hut et al., 2012). In Nile grass rats, as in other diurnal animals, masking responses to light are the reverse of those seen in nocturnal species, such that light serves to increase, rather than decrease, general activity (Shuboni et al., 2012). Additionally, many retinorecipient areas of the brain that may receive input from ipRGCs exhibit an increase in the immediate early gene product, FOS, in response to light in this species, whereas under the same conditions the response varies across regions in nocturnal mice (Shuboni et al., 2015). ipRGCs have not yet been described in the Nile grass rat but they have in a related day-active species, the Sudanese grass rat (*A. ansorgei*). Although several characteristics of these cells are similar to those reported in nocturnal rodents, some differences exist in the firing patterns of a select subtype of ipRGCs (Karnas et al., 2013a). The questions of whether ipRGCs co-store PACAP and whether the central projections of these cells are different in diurnal grass rats (either Nile or Sudanese) from those seen in nocturnal rodents have, however, not been addressed.

In the work described here we sought to determine whether retinal circuits and projections known to play a central role in masking in nocturnal rodents are present in the Nile grass rat. First, we described the melanopsin cells in the retina of the Nile grass rat and asked whether PACAP is expressed within these cells, as is the case in other species. We then characterized the distribution of central projections of PACAP-containing RGCs. To do this, we labeled PACAP within the brains of sham and enucleated Nile grass rats, and we examined overlap between PACAP fibers and retinal inputs in animals that had received intraocular injections of the anterograde tracer CT-β. Results are discussed in the context of patterns previously described in nocturnal rodents (Hannibal and Fahrenkrug, 2004; Hattar et al., 2006; Engelund et al., 2010, 2012) to determine the nature of the similarities and potential differences between the ipRGC systems of day- and night-active species.

## Materials and Methods

### Animals

Adult male Nile grass rats from a breeding colony maintained at Michigan State University were used in this study. Animals were housed in plexiglass cages (34 cm × 28 cm × 17 cm) with access to food (PMI Nutrition, Prolab RMH 2000, Brentwood, MO, USA) and water *ad libitum*, and were maintained on a 12:12 light/dark (LD) cycle (lights on at 06:00 h) unless otherwise indicated. All experiments were performed in accordance with guidelines established by the National Institutes of Health Guide for the Care and Use of Laboratory Animals and the Michigan State University Institutional Animal Care and Use Committee. All efforts were made to minimize the number of animals used in these experiments.

### Enucleations

Nine Nile grass rats were anesthetized with isoflurane and were either bilaterally (*n* = 3) or unilaterally (*n* = 3; right eye removed only) enucleated or they underwent sham surgery (*n* = 3). For enucleations, the eye was held with forceps, the optic nerve and blood vessels were severed and the eye was removed. Absorbable gelatin was inserted into the orbit and the eyelid was sealed with Vetbond (3M, St. Paul, MN, USA). Control grass rats were anesthetized but neither eye was removed. All animals were perfusion fixed 14 days after surgery and brains were collected and processed for visualization of PACAP (see below).

### Anterograde Tracing

Five intact Nile grass rats were anesthetized with isoflurane and then received 5 μl intravitreal injections through a Hamilton syringe (Reno, NV, USA) of CT-β conjugated to Alexa Fluor 488 (7 μg/μl; C-22841) in the right eye and Alexa Fluor 594 (5 μg/μl; C-22842) in the left; both were purchased from Molecular Probes (Eugene, OR, USA) and were dissolved in 2% dimethyl sulfoxide (DMSO) in 0.9% saline vehicle. Seven days following surgery, grass rats were perfusion fixed (see below).

### Tissue Collection

We collected brains from the animals described above and both retinas from three other animals maintained in a 12:12 LD cycle and four animals that were kept in constant darkness (DD) for 5 days prior to sacrifice. All of these animals were anesthetized with an intraperitoneal injection of either sodium pentobarbital (Nembutal; Ovation Pharmaceutical, Deerfield, IL, USA; 0.5 cc/animal) or urethane (Sigma–Aldrich, St. Louis, MO, USA; 1,500 mg/kg) and transcardially perfused with 0.01 M phosphate buffered saline (PBS; pH 7.4; 150–200 mL/animal) followed by Stefanini’s fixative (2% paraformaldehyde and 0.2% picric acid in 0.1 M PBS; Sigma–Aldrich, St. Louis, MO, USA; pH 7.2; 150–200 mL/animal; brain tissue), or 4% paraformaldehyde (in 0.1 M PBS; Sigma–Aldrich, St. Louis, MO, USA; 150–200 mL/animal; retinal tissue). Brains and eyes were extracted, postfixed in Stefanini’s fixative or 4% paraformaldehyde for 12–18 h, then immersed for at least 48 h in 20% sucrose solution in 0.1 M phosphate buffer (PB) and kept at 4°C. Brains were cut into coronal sections (30 μm) using a microtome [for single-label PACAP immunohistochemistry (IHC)] or a cryostat (for brains labeled with fluorescent agents). Three alternating series of sections were collected from each brain and were stored in cryoprotectant at -20°C until further processing. Retinas were orientated and dissection was performed by first removing the cornea, followed by removal of the lens. Hereafter, four cuts were made to mark the superior, nasal, inferior, and temporal quadrant and for orientation a small cut was made in the superior quadrant. The eye was then held in place by needles and the vitreous was gently removed with forceps and filter paper. After gentle dissection along the ora serrata and cut of the optic nerve, the retina was removed and kept in cryoprotectant solution (30% sucrose, 1% polyvinyl-pyrrolidone (PVP-40), 30% ethylene glycol, 0.05 M sodium phosphate buffer, pH 7.2) for better conservation, and thereafter stored at –20°C until immunohistochemically processed.

### Antibodies

A mouse monoclonal anti-PACAP antibody (MabJHH1; diluted 1:5) recognizing the epitope between amino acids 6–16 was used for both the brain and retinal tissue; this antibody has equal affinity for PACAP-27 and PACAP-38 (Hannibal et al., 1995) and shows no staining in brain sections from PACAP deficient mice (our own unpublished observations). Preabsorption of the PACAP antibody with PACAP (Hannibal et al., 1995) and omission of the primary/secondary antibody from the IHC procedure abolished staining. A rabbit anti-melanopsin antibody [41K7; diluted 1:5,000; (Hannibal et al., 2002)] directed against the C-terminal of melanopsin was used together with a N-terminal rabbit anti-melanopsin antibody (PAI-780, Fisher Scientific Inc., Barrington, IL, USA; 1:5,000) to stain melanopsin cells in the retina. No staining is observed with either of these antibodies in melanopsin deficient mice (own unpublished observation).

### Immunohistochemical Procedures

#### Retinas: PACAP + Melanopsin Immunofluorescence (IF)

To label both PACAP and melanopsin in the retina, double label IF was used. Tissue was rinsed with 0.25% Triton-X-100 (TX) in 0.01 M PBS between all steps of the procedure, and all incubations included 0.25% TX and 0.25% bovine serum albumin (BSA). All rinses and incubations occurred at room temperature, unless otherwise noted. Retinas were first treated with antigen retrieval (AR) solution in citrate buffer (pH 6.0, Sigma–Aldrich, St. Louis, MO, USA) at 80°C for 1.5 h. Next, tissue was pre-incubated with 1% H_2_0_2_ in 0.01 M PBS for 10 min, blocked with 5% normal donkey serum for 20 min, and then incubated with the PACAP antibody for 72 h at 4°C. Retinas were then incubated in the secondary antibody, biotinylated donkey anti-mouse (1:800 for PACAP; Jackson, 715-065-151) over night at 4°C. Next, tissue was incubated in avidin-biotin complex (ABC) solution (0.9% each of avidin and biotin solutions) for 30 min, biotinylated tyramide (1:50; PerkinElmer, SAT700001EA) for 1 h, and finally Alexa Fluor 488-conjugated streptavidin (1:500 for PACAP; Jackson, 016-540-084). Hereafter, tissue was placed in 1% H_2_0_2_ in 0.01 M PBS for 15 min, washed in PBS and incubated in the mixture of N- and C-terminal melanopsin antibodies for another 72 h at 4°C. After a rinse, the retinas were then incubated in Envision reagent (1:2; Dako, K4002) overnight and visualized by tyramide conjugated Alexa Fluor 594 (Molecular Probes, Eugene, OR, USA).

#### Brain: PACAP

The IHC procedure used for single labeling of PACAP in the brain was like that used for PACAP in the retina with the following exceptions: the AR step was not included, incubation with the PACAP antibody was for 48 h, the concentration of the biotinylated tyramide in which sections were incubated was 1:100, and after the 30 min incubation in ABC solution, sections were rinsed two times in 0.25% TX in 0.01 M PBS, then placed in Tris buffer (pH 7.6) for 10 min, preincubated in 0.06% diaminobenzidine (DAB, Sigma–Aldrich) in Tris buffer for 30 s and reacted with 0.01% hydrogen peroxidase for 2.5 min.

Immunofluorescence (IF) was used to label PACAP in one series of brain sections from each of the CT-β injected animals. The procedure was similar to that used for PACAP + melanopsin IF, except that incubation of the antibodies were somewhat shorter (48 h in the PACAP antibody and 1 h in the secondary), and Cy5-conjugated streptavidin (Jackson, 016-170-084) was used instead of Alexa Fluor 488-conjugated streptavidin.

### Photomicrographs

Images of DAB stained tissue were taken with a digital camera (MBF Bioscience Inc., 2007) attached to a Zeiss light microscope (Axioskop 2 Plus, Carl Zeiss, Göttingen, Germany), while fluorescent images were obtained using an iMIC confocal microscope (Till Photonics, FEI, Germany) equipped with appropriate filter settings for detecting DAPI, Cy2/Alexa Fluor 488, Texas Red/Alexa Fluor 561/594, and Cy5. Determination of whether PACAP and melanopsin were present in a single cell was done with a co-localization plug-in module in ImageJ/Fiji software (version. 1.47q, NIH, USA) in which the points of two 8-bit images with both antigens appeared white (we used default value = 255). Pixels were considered to reflect co-localization of the antigens if their intensities were higher than the threshold of their respective channels (we used a threshold set at 50–100 depending on the background noise) and if the ratio of their intensity was higher than the ratio setting value (we used the default set at 50%). Melanopsin/PACAP cell counts were performed on areas from six pieces of the same retina in which cells were stained well for both PACAP and melanopsin; areas in which one or both immunoreactions were insufficient were excluded. The sizes of the pieces ranged from 0.65 to 8.7 mm^2^ and represented both the central and peripheral retina. The retina was photographed with an iMIC confocal microscope (Till Photonics, FEI, Germany) using the wide field camera and 10× objective. Photographs were taken of each part of retina and stitched together using the LA Stitch plug-in in Fiji software (version 1.47q, NIH, USA) to create an image of the entire retina. Each of these was then analyzed using the cell counter plug-in Fiji to mark and count which cells contained only PACAP, only melanopsin, or both. Counts of melanopsin-stained cells were performed on retinas from animals either maintained in LD or kept in DD for 5 days, since a previous report in Brown Norwegian rats indicated that melanopsin expression is highest in animals maintained in constant darkness (Hannibal et al., 2013). The entire retina was photomicrographed with the iMIC confocal microscope with five stacks separated by 8 μm (*Z*-axis = 40 μm) of the entire retina covering the ganglion cell layer (GCL) until the inner nuclear cell layer (see **Figure 2**). After stitching all Z-stacks together using the LA Stitch plug-in in Fiji software (version 1.47q, NIH, USA), melanopsin cell subtypes, as defined previously in mouse retina (reviewed in Fox and Guido, 2011; Schmidt et al., 2011, and Cui et al., 2015), were counted using the 3D cell counting module in Fiji. Retinal projection (fluorescent CT-β and PACAP staining) images were obtained by the iMIC confocal microscope using filter settings for Alexa Fluor 488, 595, and 647 and images were stitched together by using Fiji with the plates being combined in Illustrator CS4 after adjusting in Photoshop CS4 (Adobe, San Jose, CA, USA). All images were adjusted for brightness and contrast, as well as for size.

## Results

### Melanopsin in the Nile Grass Rat Retina

**FIGURE 1:**
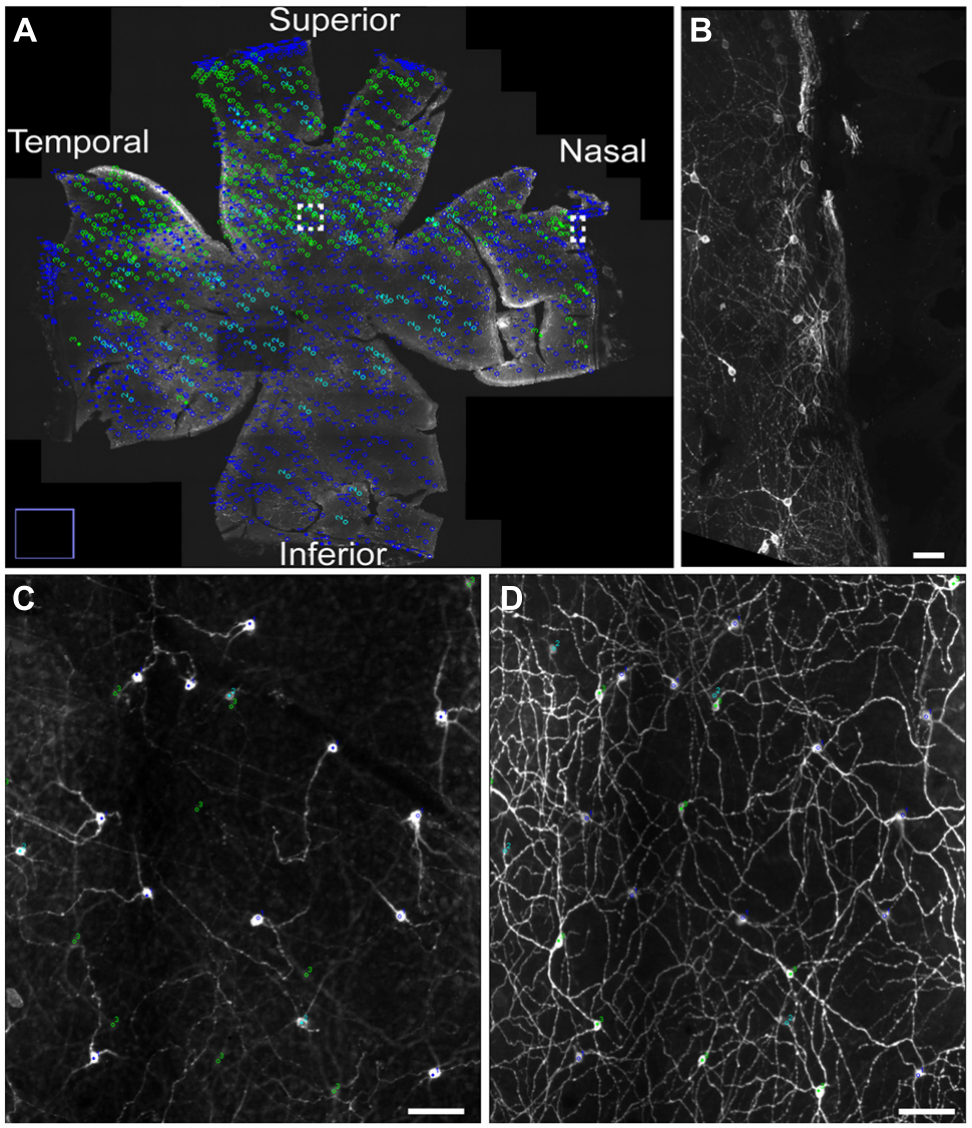
**The distribution of different subtypes of intrinsically photosensitive retinal ganglion cells (ipRGCs) across the Nile grass rat retina.** M1 and M3 ipRGCs (dark blue) and M2 (light blue) cells are evenly distributed across the retina, whereas displaced M1 cells (green) are located primarily in its superior region **(A)**. A higher power image of M1 cells is shown in **(B)**, which represents the boxed area in the nasal region of **(A)**. Melanopsin cells in the superior aspect of the retina (boxed region in **A**) are seen in the ganglion cell layer (GCL; **C**) and in the inner plexiform layer (IPL; **D**), where a dense plexus of melanopsin-containing fibers can also be seen. Scale bars: **(B–D)** = 50 μm

**FIGURE 2:**
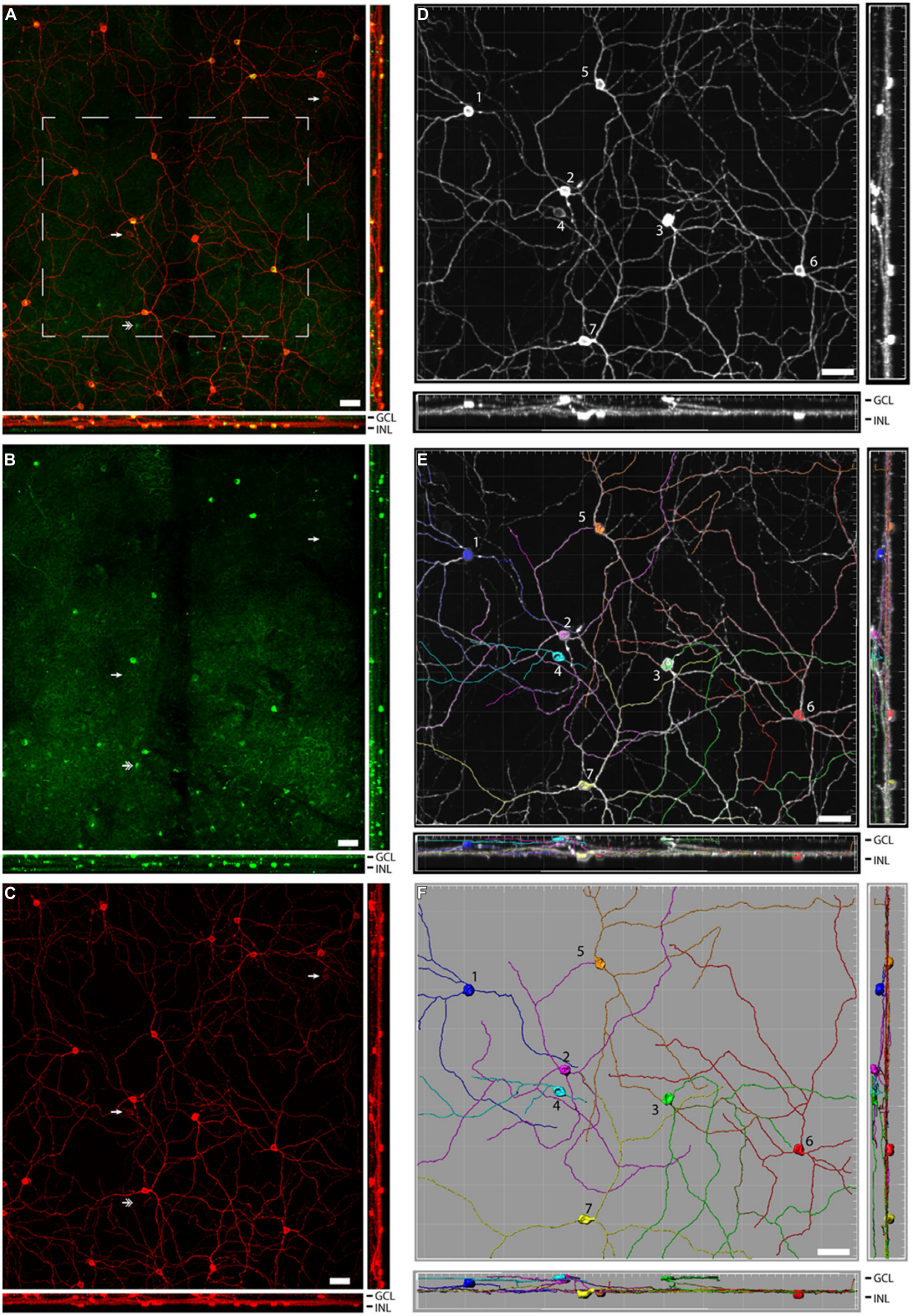
**Pituitary adenylate cyclase-activating polypeptide (PACAP) is found in several subtypes of melanopsin cells in the Nile grass rat retina.** An extended view across the Z-stack of melanopsin (red) and PACAP (green; overlay in yellow) in the retina is depicted in **(A)**, while single labels for PACAP and melanopsin are depicted in **(B,C)**, respectively. In **(A–C)**, the two single arrowheads indicate two melanopsin-expressing cells that do not express PACAP, while the double arrowhead indicates a PACAP-expressing cell that does not express melanopsin. Extended views of the melanopsin cells are shown before **(D)** and after **(E,F)** analysis of the dendritic processes. In **(D–F)** the various subtypes of melanopsin-expressing cells are marked 1–6. 1 = M1, 2 and 3 = M3, 4 = M2, and 5–7 = displaced M1 cells. GCL, ganglion cell layer; IPL, inner plexiform layer; and INL, inner nuclear layer. Scale bars: 45 μm.

Melanopsin expression was examined in flat mount retinas from animal housed in a 12:12 LD cycle and from animals housed in constant darkness due to previous reports in rats showing a slight increase in melanopsin protein expression during prolonged periods in constant darkness (Hannibal et al., 2013). Melanopsin positive cells were found in the GCL and displaced in the inner nuclear layer (INL) in the superior half of the retina (**Figure 1**). Melanopsin was located mainly in the cell membrane of the soma and dendritic processes but in animals kept in constant darkness melanopsin was also found in the membrane of axons projecting to the optic nerve (**Figure 1**). As previously reported in mice (reviewed in Fox and Guido, 2011; Schmidt et al., 2011; and Cui et al., 2015), melanopsin-containing cells could be identified as being either as subtype M1 [cell soma in the GCL or in the INL and dendrites in the distal (“OFF”) sublamina of the inner plexiform layer (IPL), known as S5], M2 [weak melanopsin expression, cell soma in the GCL and dendrites in the proximal (“ON”) sublamina of the IPL, known as S1)], or M3 (cell soma in the GCL and dendritic processes in both S1 and S5), (**Figure 2**). We were unable to identify melanopsin cell types as M4 or M5 in the grass rat retina. Whereas M1 and M3 cells (∼74% of all ipRGCs), as well as M2 cells (∼6% of all ipRGCs) were scattered relatively evenly across the entire retina, displaced M1 cells (i.e., with cell bodies in INL; ∼20% of all ipRGCs) were located primarily in superior regions of the retina (**Figure 1**). Interestingly, a large number of melanopsin-expressing cells were found in of the superior, nasal, and temporal periphery of the grass rat retina (**Figures 1A,B**) as recently described in the rat (Vugler et al., 2008) and mouse (Semo et al., 2014; Valiente-Soriano et al., 2014). These cells all co-stored PACAP (see also below). Cell counts revealed that in animals housed in a 12:12 LD cycle, 1,138 ± 89 melanopsin cells (27.6 ± 1.3 cell/mm^2^) were present whereas in animals housed in constant darkness, 1,402 ± 52 melanopsin cells (32.7 ± 1.3 cell/mm^2^) were counted; this difference was not statistically significant [*t*(3) = 2.165, *p* = 0.12). However, the dendritic network appeared to be denser and axonal staining of melanopsin was present in animals housed in constant darkness.

### Melanopsin and PACAP in the Grass Rat Retina

**FIGURE 3:**
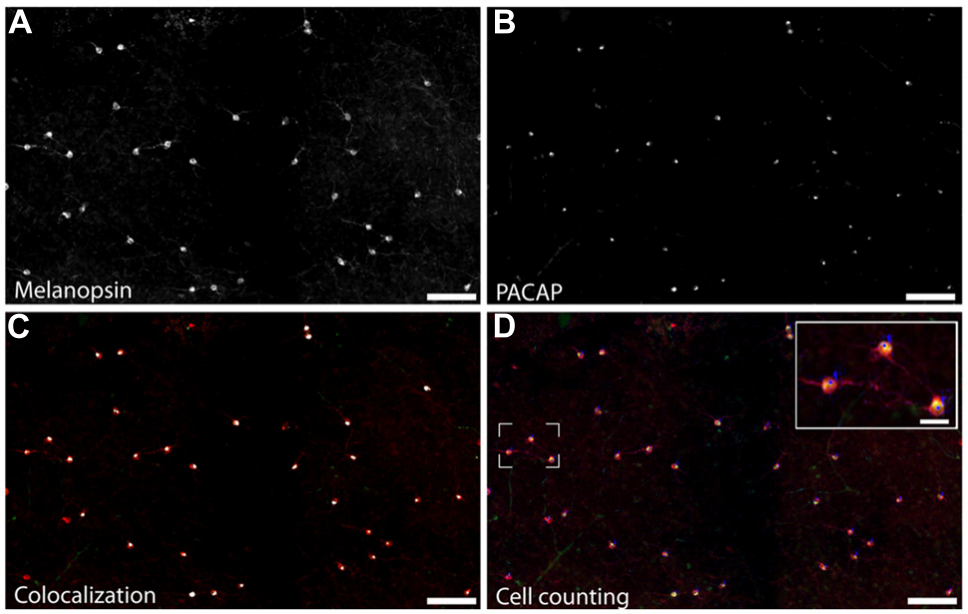
**Staining for melanopsin and PACAP reveals that they are expressed in the same cells in the Nile grass rat retina.** Photomicrographs depict melanopsin-immunoreactive (ir) cells **(A)**, PACAP-ir cells **(B)** and the overlay of staining for PACAP and melanopsin (**C**; melanopsin-ir in red and the pixel overlap of melanopsin-ir and PACAP-ir in white). Manual counts of cells expressing melanopsin and PACAP were done in Fiji **(D)**. Scale bars: **(A–D)** = 100 μm and insert in **(D)** = 30 μm.

A total of 633 cells containing melanopsin were counted in six pieces of the same retina. Of these, 555 (87.7%) co-stored PACAP (**Figures 2** and **3**); this percentage ranged from 84 to 94 across the different pieces of retina. PACAP was seen in all subtypes of melanopsin cells. A very small number of cells (only 15 out of 570, 2.6%) containing only PACAP (i.e., no melanopsin) were counted. None of the six pieces of the retina had a melanopsin cell density that was below the average density found when counting the total number of melanopsin cells. This finding in the grass rat retina was similar to that of the monkey retina (Hannibal et al., 2014), in which a very small number of cells containing melanopsin, but not PACAP, or PACAP, but not melanopsin, were found (**Figure 2**). Since we found some variation between the numbers of melanopsin/PACAP containing cells in the different pieces of retina we cannot exclude the possibility that some melanopsin cells don’t express PACAP (and vice versa) but more likely this finding is due to low levels of expression of PACAP or melanopsin in some cell types.

### PACAP Fibers in Retinorecipient Regions of the Grass Rat Brain

Pituitary adenylate cyclase-activating polypeptide-immunoreacti-vity in the grass rat brain was observed in many regions known to receive input from ipRGCs in rats (Hannibal and Fahrenkrug, 2004) and in mice (Hattar et al., 2006). The structures described below are ones that had noticeable reductions in PACAP-immunoreactive (ir) fibers following removal of the eyes and a high degree of overlap between the distributions of PACAP and CT-β-labeled fibers. These areas included the SCN, LGN, pretectum, and SC. Other hypothalamic structures, such as the ventrolateral preoptic area, subparaventricular zone, and lateral hypothalamus, are known to receive input from ipRGCs in nocturnal rats (Hannibal and Fahrenkrug, 2004) and mice (Hattar et al., 2006). However, it was difficult to determine if this was the case in grass rats, as retinal input to these areas is minimal (Todd et al., 2012; Gaillard et al., 2013) and many non-retinal PACAP-ir fibers are present throughout the hypothalamus (data not shown).

### Suprachiasmatic Nucleus

**FIGURE 4:**
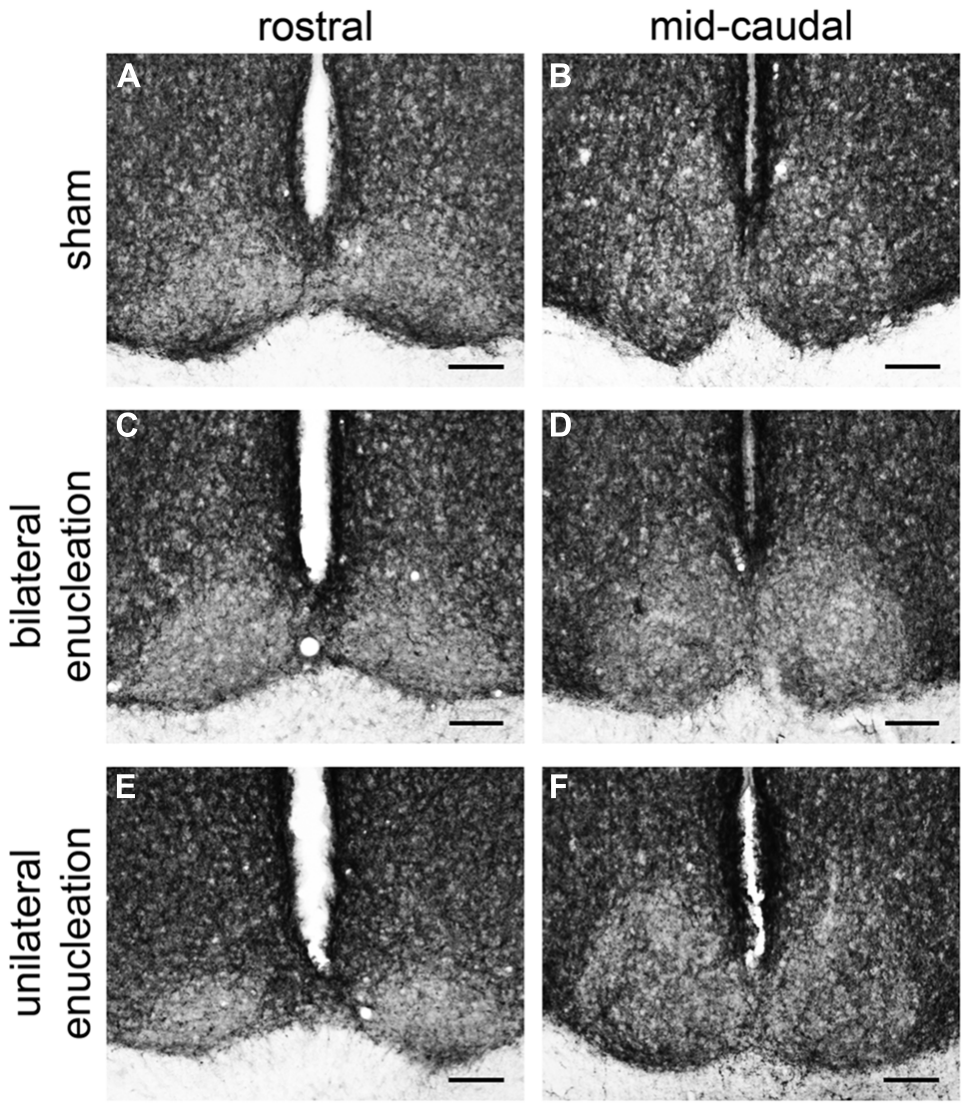
**Pituitary adenylate cyclase-activating polypeptide-immunoreactive fibers in the rostral and mid-caudal suprachiasmatic nucleus (SCN) of sham, bilaterally, and unilaterally enucleated Nile grass rats.** Sham Nile grass rats had PACAP-ir fibers in the ventral portion of the rostral SCN **(A)** and in both the dorsal and ventral part of the mid-caudal SCN **(B)**. Bilateral enucleation reduced PACAP-ir fibers across the rostrocaudal extent of the SCN **(C,D)**. Unilateral enucleation reduced PACAP-ir fibers in the SCN, but some were still present, particularly in its mid-caudal region **(E,F)**. Scale bars: 100 μm.

**FIGURE 5:**
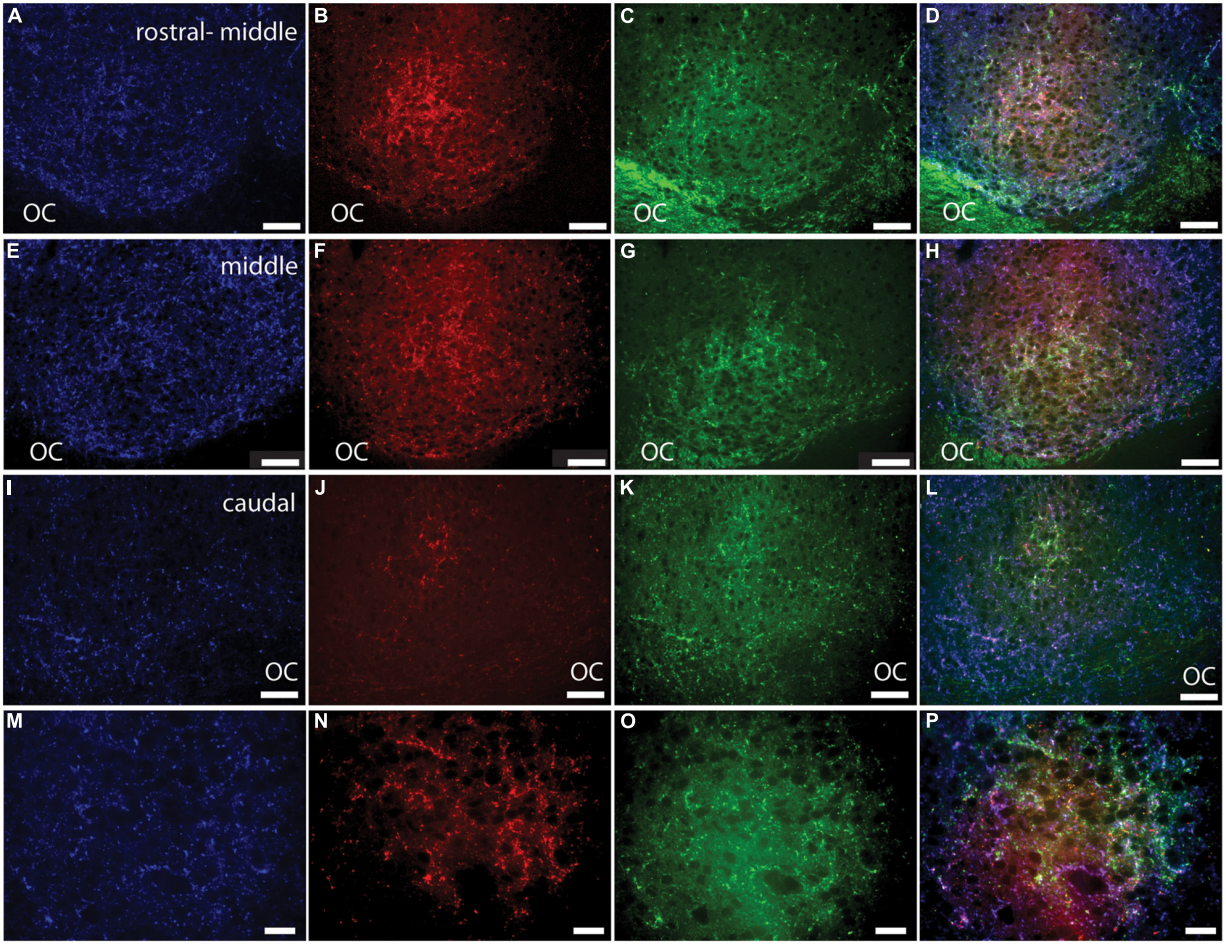
**Pituitary adenylate cyclase-activating polypeptide-immunoreactive (blue; A, E, I, M) fibers and cholera toxin subunit β (CT-β)-labeled retinal fibers from the ipsilateral (red; B, F, J, N) and contralateral (green; C, G, K, O) eye in the rostral-middle (A–D), middle (E–H), and caudal (I–L) left SCN**. An overlay of the PACAP staining and the tracers is pictured in **(D**, **H**, **L**, and **P)**. Magenta and cyan colors in (**D**, **H**, **L**, and **P**) represent the overlap of the PACAP-ir labeling and CT-β-labeled retinal fibers, each of which can be seen at all levels of the SCN. Low magnification images of various levels of the SCN are presented in **(A–L)** and higher magnification images of areas shown in **(E–H)** are illustrated in **(M–P)**. OC, optic chiasm. Scale bars: **(A–L)** = 50 μm and **(M–P)** = 20 μm.

In the rostral SCN of sham grass rats, PACAP-ir fibers were more ventrally located (**Figure 4A**), while in the mid-caudal portions of the SCN, PACAP labeling was present across the nucleus (**Figures 4B** and **5**). PACAP-ir fibers were greatly reduced in bilaterally enucleated grass rats compared to shams throughout the rostrocaudal extent of the SCN (**Figures 4C,D**). In unilaterally enucleated grass rats PACAP fiber labeling was reduced but still present (**Figures 4E,F**); the reduction was similar in the SCN ipsilateral and contralateral to the eye that had been removed, indicating that PACAP fibers from the retina have bilateral projections to the SCN. This was confirmed by the bilateral tracing experiments, which also show that the majority of PACAP-ir nerve fibers in the SCN originate from the retina (**Figure 5**).

### Lateral Geniculate Nucleus

**FIGURE 6:**
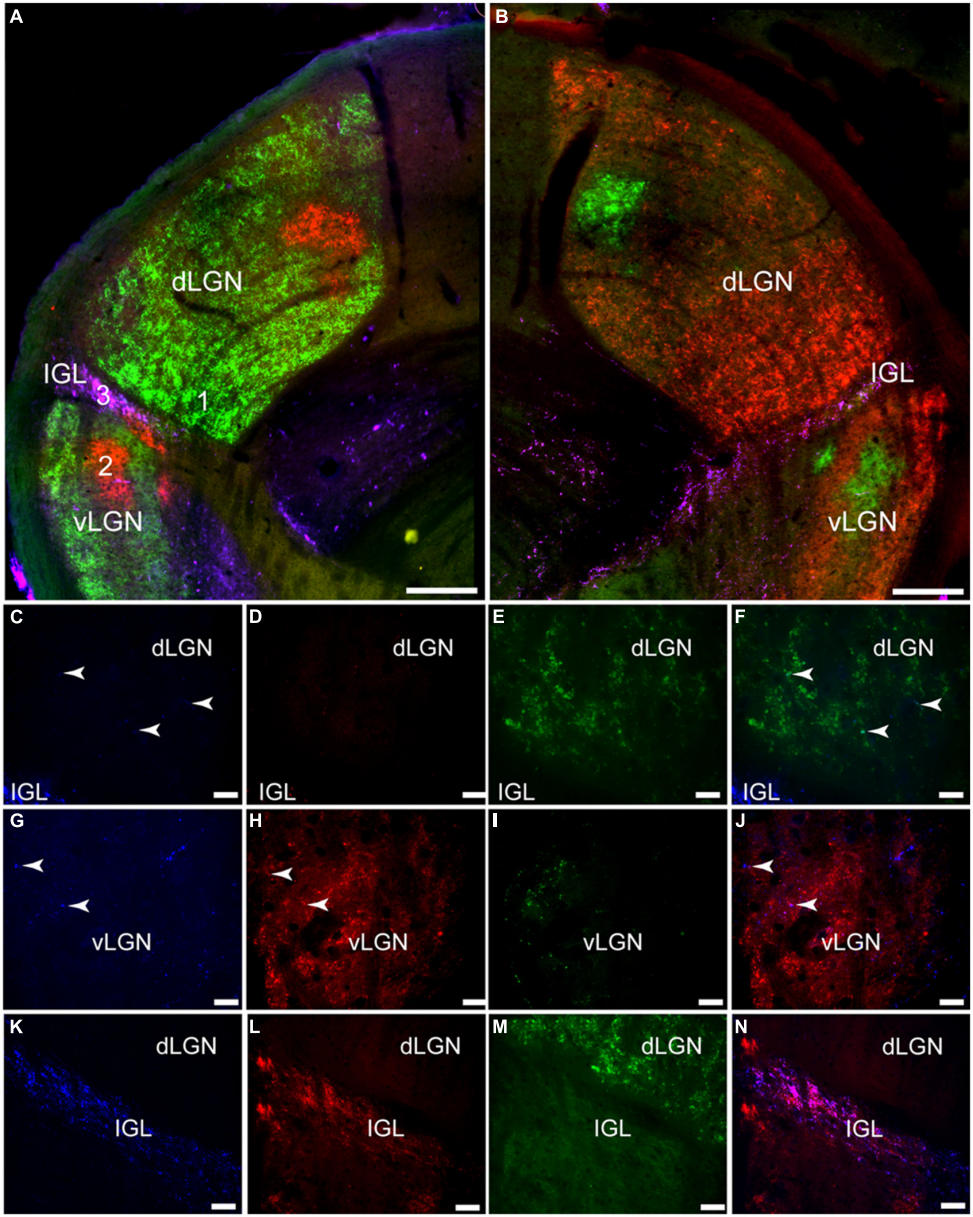
**Pituitary adenylate cyclase-activating polypeptide-immunoreactive (blue) fibers and CT-β-labeled retinal fibers from the left (red) and right (green) eye in the lateral geniculate complex (LGN) of the Nile grass rat.** Low magnification images of the left LGN are shown in **(A)** and of the right in **(B)**. Higher magnification images in **(C–N)** depict the numbered regions in (**B**; 1 = **C–F**, 2 = **G–J**, 3 = **K–N**). Few PACAP-ir fibers were seen in the dorsal LGN (dLGN; **C**) where inputs from the contralateral eye were present **(E,F)**; some PACAP-ir fibers were present in the ventral LGN (vLGN; **G**) where retinal fibers from the ipsilateral eye were concentrated (vLGN, **H** and **J**). A dense plexus of PACAP-ir fibers could be seen in the intergeniculate leaflet (IGL; **K**); there was a high degree of overlap between them and CT-β-labeled retinal fibers **(L–M)**. Scale bars: **(A,B)** = 200 μm, **(C–J)** = 20 μm, and **(K–N)** = 40 μm.

**FIGURE 7:**
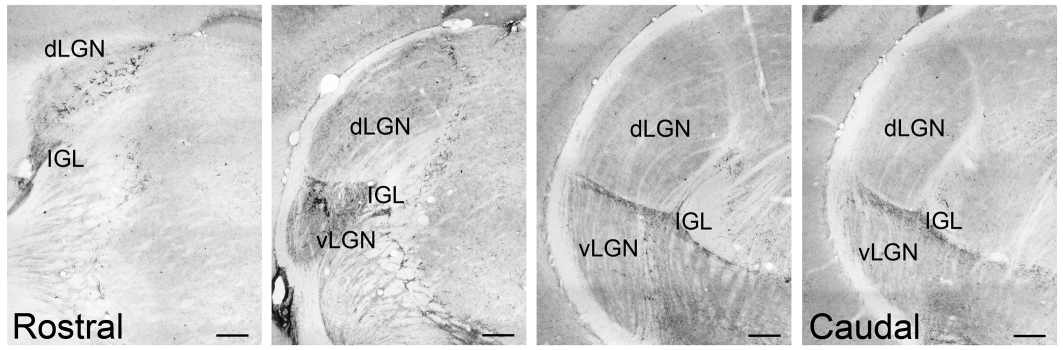
**Pituitary adenylate cyclase-activating polypeptide-immunoreactive fibers across the rostrocaudal extent of the LGN of the Nile grass rat.** PACAP-ir fibers were present in the dorsal aspect of the LGN (dLGN) only in its most rostral region, whereas they were observed in the full rostrocaudal extent of the IGL; very few fibers were seen in ventral LGN (vLGN). Scale bars: 200 μm.

**FIGURE 8:**
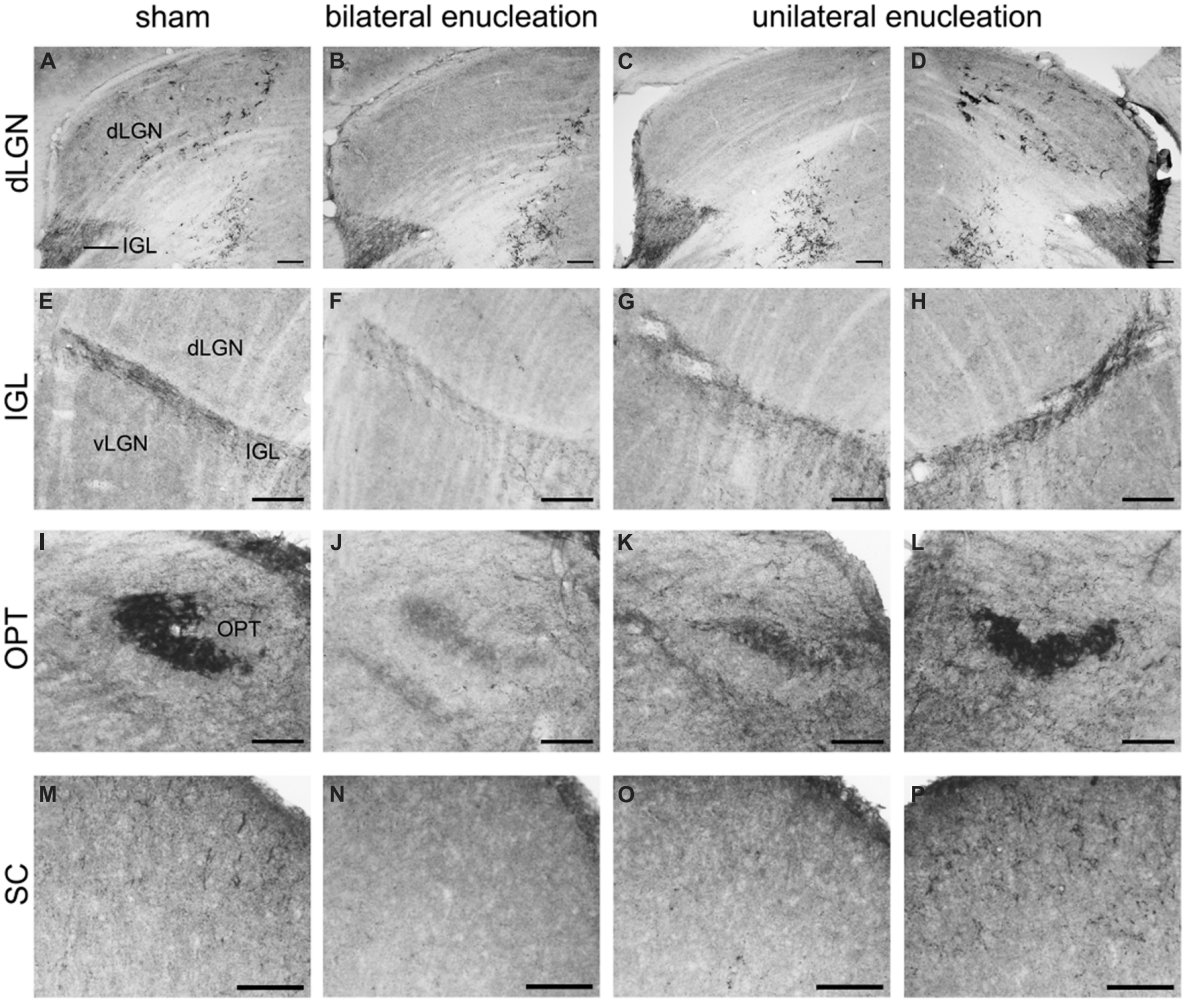
**Pituitary adenylate cyclase-activating polypeptide-immunoreactive (ir) fibers in the dLGN, IGL, olivary pretectal nucleus (OPT), and superior colliculus (SC) of sham, bilaterally, and unilaterally enucleated Nile grass rats.** PACAP-ir fibers were observed in the dLGN **(A)**, IGL **(E)**, OPT **(I)**, and SC **(M)** of sham Nile grass rats; PACAP-ir labeling was greatly reduced in all of these regions in bilaterally enucleated Nile grass rats (**B**, **F**, **J**, **N**) indicating that most of these fibers come from the retina. Unilateral enucleation reduced PACAP-ir fibers more in the regions contralateral to the eye that was removed (**C**, **G**, **K**, and **O**) than ipsilateral to it (**D**, **H**, **L**, and **P**), indicating that many (though not all) of these fibers are crossed. Scale bars: 100 μm.

The bilateral retinal tracing demonstrates a distinct pattern of projections from the eye to this part of the grass rat brain (**Figure 6**). Most of these fibers come from the contralateral eye (**Figure 6**), as previously shown in these animals (Gaillard et al., 2013). Fibers labeled by CT-β injections into the ipsilateral eye were mostly concentrated in a distinct region of the central part of the dorsal LGN (dLGN) and of the ventral LGN (vLGN) and because of this, such ipsilateral fibers clearly demarcate the intergeniculate leaflet (IGL). Most of the dLGN was devoid of PACAP, but some thick PACAP-ir fibers were found in its most rostral region (**Figures 7** and **8A**); this pattern was similar to that of melanopsin cell projections to this area in mice (Hattar et al., 2006; Ecker et al., 2010). These PACAP-ir fibers were eliminated with bilateral removal of the eyes (**Figure 8B**) and were only present in the dLGN contralateral to the remaining eye in unilaterally enucleated animals (**Figures 8C,D**) indicating that melanopsin/PACAP projections from the retina to this region are completely crossed. Very few PACAP-ir fibers were observed in mid-caudal dLGN (**Figures 6** and **7**); however, those seen were overlaid with CT-β-labeled retinal fibers from the contralateral eye (**Figures 6C–F**).

Relatively few PACAP-ir fibers were found in the ventral portion of the vLGN (**Figures 6–8**). Their distribution in this region overlapped to some extent with that of CT-β-labeled retinal fibers emanating from the ipsilateral eye (**Figures 6G–J**). A few PACAP-ir cells were present in the vLGN. These cells were in the outer boundaries (most medial and lateral portion) of the vLGN and were relatively sparse in the central region.

### Intergeniculate Leaflet

Pituitary adenylate cyclase-activating polypeptide-immunoreactive fibers densely innervated the IGL and spanned across its full rostrocaudal extent (**Figure 7**). Although PACAP-ir labeling was not reduced in bilaterally enucleated grass rats in the most rostral portion of the IGL (**Figures 8A,B**) PACAP-ir in the mid-caudal region of the IGL was substantially reduced after removal of both of the eyes (**Figures 8E,F**). In unilaterally enucleated animals PACAP-ir was diminished slightly more on the side of the brain contralateral to the eye that had been removed (**Figures 8G,H**), indicating that many of these fibers are crossed; however, many fibers were still present on both sides of the IGL, indicating that PACAP-ir projections from the retina to the IGL are bilateral. This was supported using bilateral tracing, which demonstrated a high degree of overlap between the retinal tracers from the two eyes with PACAP fibers in the IGL (**Figures 6K–N**). Similar to the vLGN, a few PACAP-ir cells were found within the IGL (**Figures 8E–H**).

### Pretectum

**FIGURE 9:**
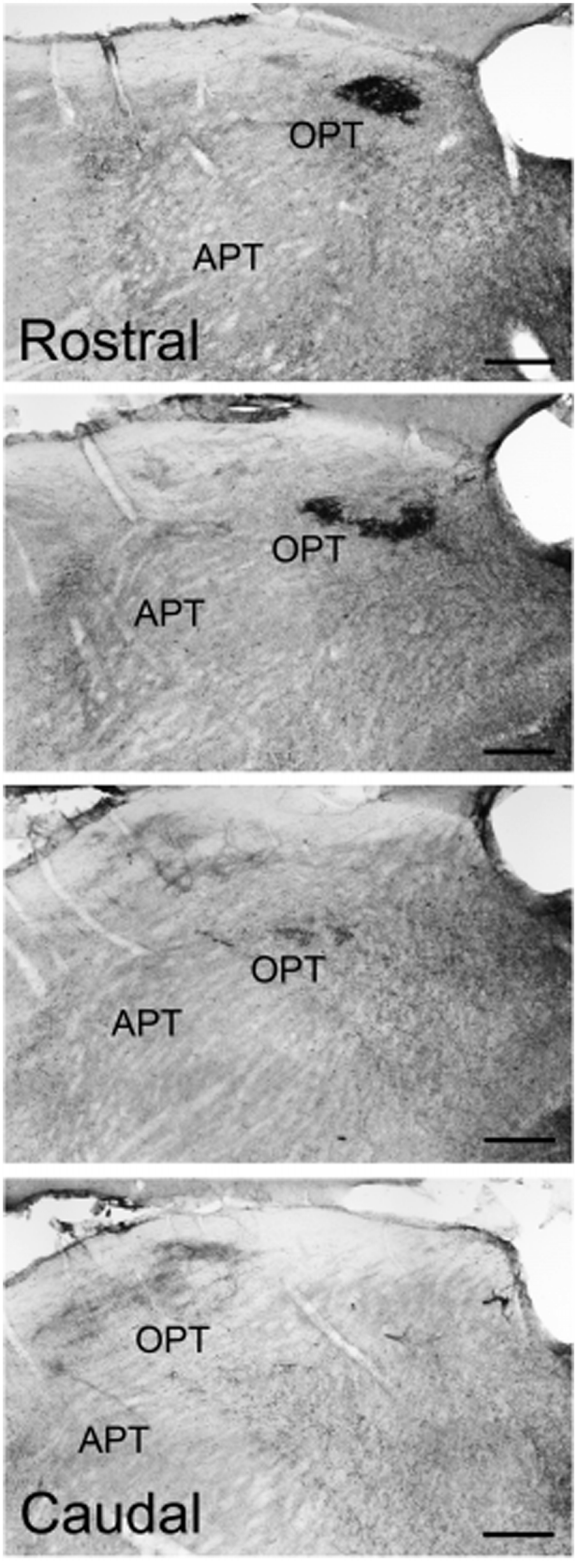
**Pituitary adenylate cyclase-activating polypeptide-immunoreactive fibers across the rostrocaudal extent of the OPT of the Nile grass rat.** Intense PACAP fiber labeling was seen in the most rostral regions of the OPT, while very little was observed in the more caudal regions of the OPT. APT, anterior pretectal nucleus. Scale bars: 20 μm.

**FIGURE 10:**
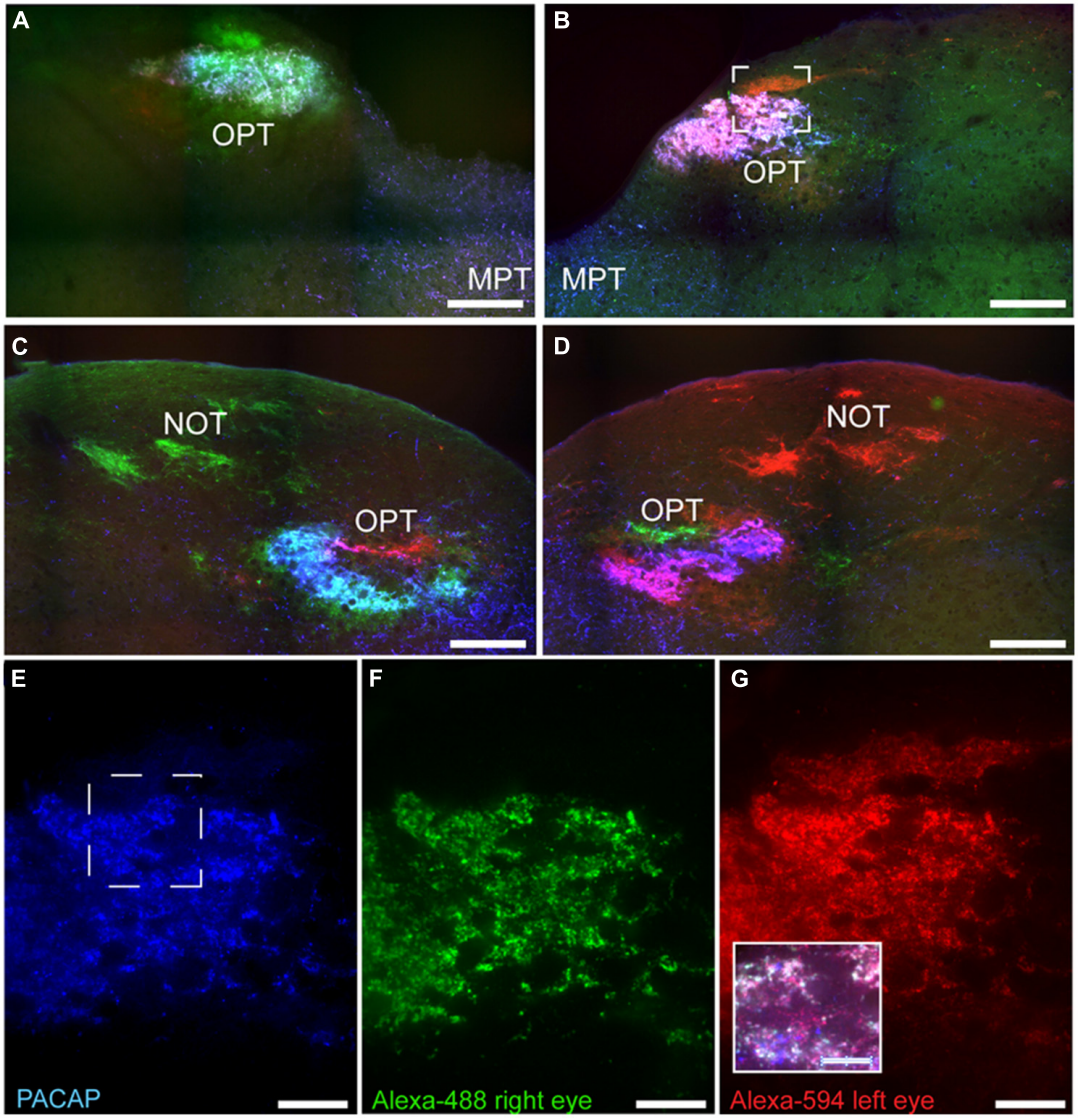
**Pituitary adenylate cyclase-activating polypeptide-immunoreactive (blue) fibers and CT-β-labeled retinal fibers from the left (red) and right eye (green) eye in the OPT of the Nile grass rat.** PACAP-ir fibers were concentrated in regions with CT-β-labeled retinal fibers from the contralateral eye; this was the case both in the most rostral pole of the OPT **(A,B)** and immediately caudal to this region **(C,D)**. High magnification images of PACAP-ir fibers **(E)** and retinal fibers from the right **(F)** and left **(G)** eye were taken from the boxed area shown in **(B)**. Within this most rostral portion of the OPT the area in which PACAP-ir fibers are most concentrated also receives the most input from each eye. The insert in **(G)** illustrates an overlay of all three labels present in the boxed area shown in **(E)**. MPT, medial pretectal nucleus; NOT, nucleus of the optic tract. Scale bars: **(A–D)** = 250 μm, **(E–G)** = 50 μm, and insert in **(G)** = 25 μm.

Pituitary adenylate cyclase-activating polypeptide-immunoreactive fibers within the pretectum were concentrated in the olivary pretectal nucleus (OPT; **Figures 8I** and **9**). Although the rostrocaudal extent of the grass rat OPT, as defined by retinal projections, is extensive (∼1,080 μm; Gaillard et al., 2013), PACAP was only observed in its most rostral region (**Figure 9**). Most PACAP-ir fibers in the OPT originated from the retina, as bilateral enucleation greatly reduced their density (**Figure 8J**). PACAP-ir fibers were observed in both the left and right OPT in unilaterally enucleated grass rats, but more were present in the OPT ipsilateral to the eye that had been removed, indicating that most fibers are crossed (**Figures 8K,L**). This observation was supported by the high degree of overlap between CT-β positive retinal fibers and PACAP-ir fibers within the OPT (**Figure 10**), especially at its most rostral pole (**Figures 10A,B,E–G**). A distinct distribution of ipsilateral and contralateral innervation was revealed by the bilateral tracer injections which demonstrated that contralateral projections target the central and ventral OPT, while input from the ipsilateral eye is concentrated in the peripheral part of the dorsal region of the OPT (**Figures 10C,D**). Most prominent co-localization between PACAP and Ct-β was found in the central and ventral part of the OPT.

**FIGURE 11:**
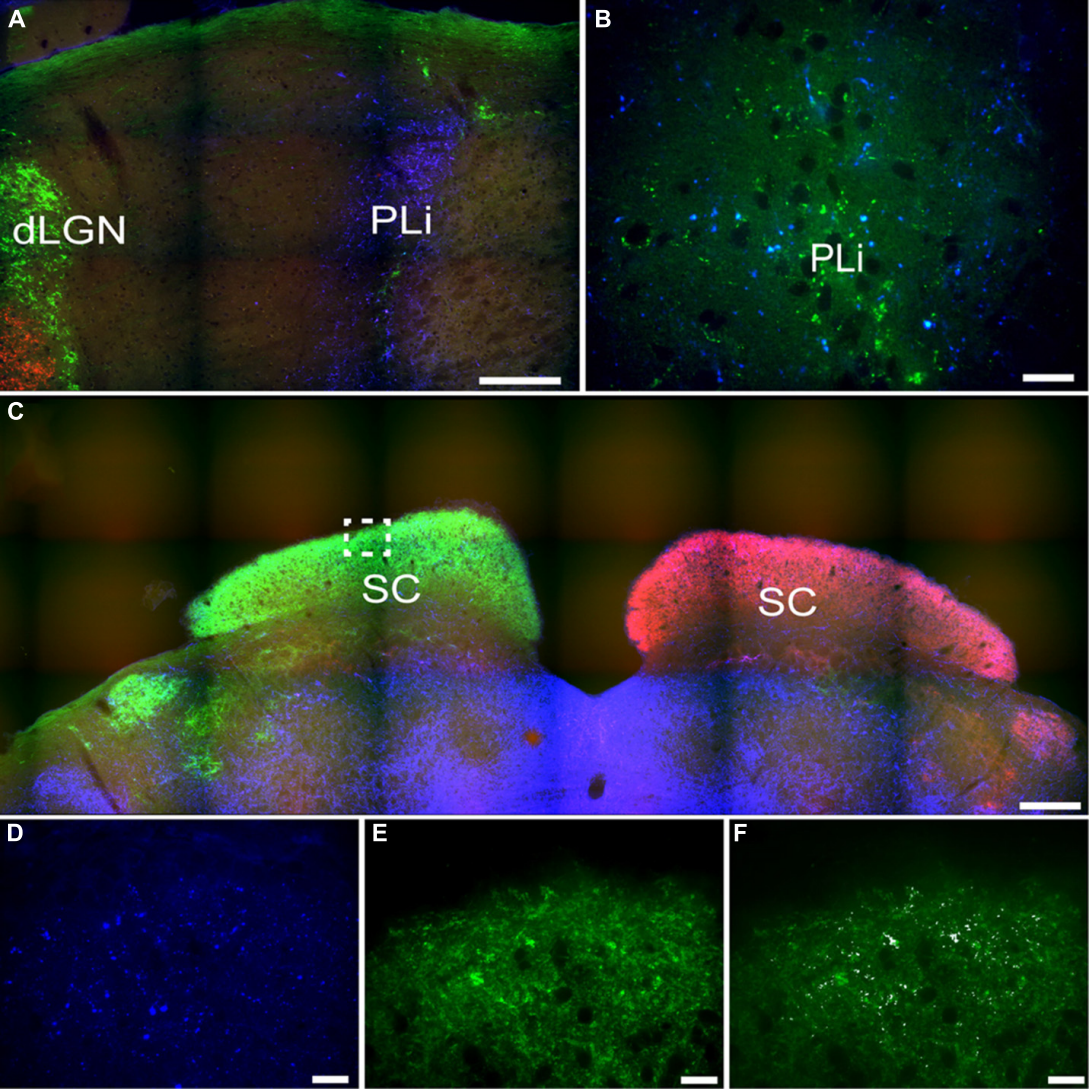
**Pituitary adenylate cyclase-activating polypeptide-immunoreactive (blue) and CT-β-labeled retinal fibers from the left (red) and right eye (green) eye in the posterior limitans (PLi) and SC.** Many PACAP-ir fibers were present in the PLi **(A)**, while a small number of these fibers also overlaid with retinal fibers from the contralateral eye (green; **B**). The SC is heavily innervated by retinal fibers from the contralateral eye **(C)** and some of these fibers also overlay with PACAP-ir labeled fibers (**D–F** higher magnification of boxed area in **C**; **D** = PACAP staining, **E** = retinal fibers, **F** = overlay with co-localization shown in white). Scale bars: **(A)** = 150 μm, **(B)** = 20 μm, **(C)** = 300 μm, and **(D–F)** = 20 μm.

Another pretectal structure that contained PACAP-ir fibers was the posterior limitans (PLi). Although PACAP-ir did not seem to be reduced in enucleated grass rats, overlap between PACAP-ir and the retinal tracer from the contralateral eye indicate that a few of these fibers originate from the retina (**Figures 11A,B**). However, many PACAP-ir fibers in the PLi did not overlap with the retinal tracers, indicating that many of the PACAP fiber within this area do not originate in the retina.

### Superior Colliculus

A few intensely labeled fibers were found in the SC of intact grass rats (**Figure 8M**) but they were absent in bilaterally enucleated ones (**Figure 8N**). In unilaterally enucleated animals PACAP-ir labeled fibers were denser on the side contralateral to the remaining eye and virtually eliminated on the ipsilateral side (**Figures 8O,P**). Similarly, there was a high degree of overlap between PACAP-ir fibers and CT-β-labeled retinal fibers from the contralateral eye (**Figures 11C–F**), again demonstrating the crossed nature of PACAP-containing retinal fibers in this region. As found in other rodents, PACAP-expressing neurons were found in the deep layers of the SC (not shown).

## Discussion

### ipRGCs and PACAP in the Retina of Nile Grass Rats

Intrinsically photosensitive retinal ganglion cells are required for the acute inhibition of activity by light in nocturnal rodents and are of special interest in Nile grass rats because light elicits the opposite response in these animals (Shuboni et al., 2012). Here, we found that the retina of the Nile grass rat expresses melanopsin-containing ganglion cells that represent ipRGCs. An increased number of melanopsin cells and enhanced melanopsin-immunoreactivity in dendritic processes were seen in grass rats kept in constant darkness compared to those maintained in a 12:12 LD cycle, though this difference did not reach statistical significance (*p* = 0.12). This trend, however, followed the same pattern as that seen with melanopsin-immunoreactivity in brown Norwegian rats (Hannibal et al., 2013) and albino Wistar rats (Hannibal et al., 2005). Interestingly, the total number of ipRGCs, even in constant darkness, was somewhat low in our grass rats compared to the closely related diurnal Sudanese grass rat (Karnas et al., 2013a). The reason for the difference is unclear, but it may be related to the fact that our Nile grass rats were derived from a population living 3° South of the equator (Katona and Smale, 1997; McElhinny et al., 1997; Blanchong and Smale, 2000), whereas the Sudanese grass rats were derived from animals trapped 12° North of the equator (Challet et al., 2002; Cuesta et al., 2009).

Different subtypes of ipRGCs are defined mainly in mice (Baver et al., 2008; Schmidt and Kofuji, 2009; Berson et al., 2010; Ecker et al., 2010) and rats (Esquiva et al., 2013; Reifler et al., 2015) on the basis of a number of characteristics, including their sizes and the location of their dendrites within the retina. The three major subtypes seen in other species (M1–M3) were present in the Nile grass rat retina. Most M1–M3 cells were distributed evenly across the retina, but the density of displaced M1 cells (with cell bodies in the INL) was greater in the superior region (**Figure 1**); a larger number of ipRGCs are observed in the superior retina of nocturnal rats (Hannibal et al., 2002; Hattar et al., 2002; Esquiva et al., 2013; Galindo-Romero et al., 2013). In contrast, uniform distributions of all types of ipRGCs are seen in the mouse (Berson et al., 2010; Brown et al., 2010), hamster (Bergstrom et al., 2003; Morin et al., 2003), and human (Hannibal et al., 2004) retina, while macaques have higher numbers of ipRGCs in the central compared to the peripheral retina (Dacey et al., 2005; Hannibal et al., 2014). Many large clusters of ipRGCs were also observed near the retinal ciliary marginal zone of the superior, nasal, and temporal retina of Nile grass rats (**Figure 1**). Similar clusters are reported in the peripheral region of either the superior (Vugler et al., 2008; Valiente-Soriano et al., 2014) or nasal region of the retina (Semo et al., 2014) of nocturnal rodents. These cells have been shown to project to the ciliary body where they control the intrinsic PLR in mice (Semo et al., 2014). Interestingly, in the Nile grass rat, all of the melanopsin cells clustered in the peripheral retina also contained PACAP. Thus, not only does it seem likely that these cells play a similar role in the intrinsic PLR in Nile grass rats, but it also suggests that PACAP may serve as a neurotransmitter regulating this reflex in the retina.

The relative numbers of different subtypes of ipRGCs vary somewhat from species to species. In Nile grass rats, 94% were M1 and M3 cells and only 6% were of the M2 subtype, whereas in Sudanese grass rats 25% are of the M2 subtype (Karnas et al., 2013a). In that species M1 cells represent 74% of the total (Karnas et al., 2013a), which is higher than what has been reported in nocturnal rodents (Berson et al., 2010; Schmidt and Kofuji, 2011; Karnas et al., 2013b). Another difference between Nile and Sudanese grass rats is that the number of displaced M1 cells was proportionally higher in the former (20% of ipRGCs) than in the latter (<1% of ipRGCs), (Karnas et al., 2013a). Interestingly, in humans, as in Nile grass rats, the number of ipRGCs that are displaced is disproportionately high (50% of the total; Hannibal et al., 2004).

Pituitary adenylate cyclase-activating polypeptide has been identified in melanopsin-containing cells in a number of species and is present in axons projecting into the brain, where it plays a neuromodulatory role (Hannibal et al., 2002, 2004, 2014; Bergstrom et al., 2003; Hannibal and Fahrenkrug, 2004; Hannibal, 2006). Here, we found that 84–94% (average 87.7%) of the ipRGCs of Nile grass rats also expressed PACAP (**Figures 2** and **3**). The number of RGCs co-storing PACAP and melanopsin was slightly lower compared to that previously reported in the rat, hamster, monkey, and human (Hannibal et al., 2002, 2004, 2014; Bergstrom et al., 2003). Since PACAP was found in all subtypes of melanopsin cells it seems unlikely that the PACAP-negative/melanopsin-positive cells or PACAP-positive/melanopsin-negative cells represent distinct populations of cells. More likely, this reflects a difference in expression of either melanopsin or PACAP since both genes are regulated by light and by a circadian clock (Hannibal et al., 2005, 2013) and/or a technical limitation on our ability to detect very low levels of melanopsin/PACAP.

### ipRGC Projections

**FIGURE 12:**
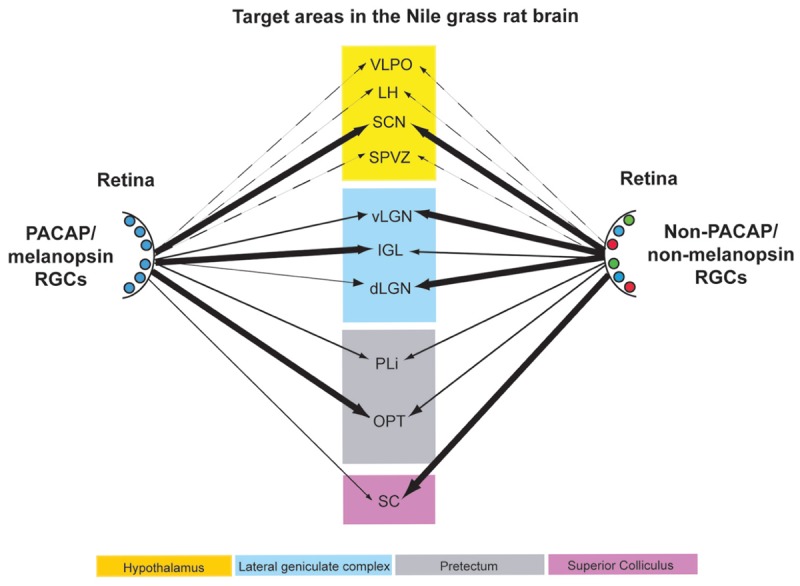
**Schematic diagram of the retinal projections from PACAP/melanopsin-containing RGCs (left) and RGCs not containing PACAP/melanopsin (right) in the Nile grass rat brain.** The thickness of the arrows roughly indicates the density of the innervation to the various brain regions listed. Some projections of each type that are presumed to exist in other hypothalamic regions are presented with dashed lines. VLPO, ventrolateral preoptic area; LH, lateral hypothalamus; SCN, suprachiasmatic nucleus; SPVZ, subparaventricular zone; vLGN, ventral lateral geniculate nucleus; IGL, intergeniculate leaflet; dLGN, dorsal lateral geniculate nucleus; PLi, posterior limitans; OPT, olivary pretectal nucleus; SC, superior colliculus.

It is not possible to use transgenic procedures to aid in identification of axons within the grass rat brain that emanate from ipRGCs, as it is in mice, but our data suggest that PACAP may be used to do this instead. Specifically, by comparing distributions of PACAP-ir fibers in intact and enucleated animals, and by examining direct overlays of retinal and PACAP-ir fibers, we can make reasonable inferences about where the ipRGCs project in Nile grass rats. Using these approaches we found evidence of ipRGC projections in many regions of the Nile grass rat brain that likely play a role in masking, including the SCN, LGN, pretectum, and SC (**Figure 12**). These areas are all known to receive such input in other species (Morin et al., 2003; Hannibal and Fahrenkrug, 2004; Dacey et al., 2005; Hattar et al., 2006; Brown et al., 2010; Ecker et al., 2010; Hannibal et al., 2014), though there is some variation in its patterns and densities. Many other hypothalamic areas receive input from ipRGCs in nocturnal rodents (Hannibal and Fahrenkrug, 2004; Hattar et al., 2006), and are of interest as they show patterns of activity that are not the same in those animals and grass rats (reviewed in Smale et al., 2008). However, retinal input to these areas is limited and PACAP fibers emanating from other sources are dense, making it impossible for us to determine if ipRGCs project to these regions.

### Suprachiasmatic Nucleus

We obtained evidence of bilateral projections from ipRGCs to the SCN in the Nile grass rat. Specifically, PACAP-immunoreactivity was reduced but still present bilaterally in the SCN following unilateral enucleation, and was almost completely eliminated when both eyes were removed. Bilateral tracing experiments also indicated that the major source of PACAP fibers within the SCN is the retina, and that PACAP cells within each eye project to both to the left and right SCN (**Figures 4** and **5**). The few non-retinal PACAP-ir fibers within the SCN may come from the IGL or vLGN, as there are weakly stained PACAP-ir neurons in both areas in grass rats (**Figure 8**). Such cells have not been reported in Wistar rats (Hannibal, 2002), raising the intriguing possibility that they may play a role in mediation of differences in the behavioral responses to light of these two species. It is not possible to tell which subtypes of ipRGCs project to the SCN of Nile grass rats. In mice, however, both M1 and non-M1 cells innervate this nucleus (Ecker et al., 2010) and most of the input comes from M1 cells (Baver et al., 2008).

Intrinsically photosensitive retinal ganglion cells projections to the SCN of nocturnal mice carry information about light that comes both from its effect on the melanopsin protein and from cone/rod-driven pathways that converge on the ipRGCs and this pathway mediates the entraining effects of light on circadian rhythms (Goz et al., 2008; Guler et al., 2008; Hatori et al., 2008). The ipRGCs are likely to convey photic information via similar mechanisms to the SCN of Nile grass rats, as photic responses of the circadian system are very similar in diurnal and nocturnal species (reviewed in Smale et al., 2003). However, some differences in this system may exist in relation to the role that it plays in mediation of the more acute (“masking”) responses to light, which are not the same in diurnal and nocturnal species. The extent to which masking depends on the SCN has been debated, but in the most recent and thorough study, SCN lesions in hamsters abolished light-induced suppression of activity (Li et al., 2005). However, preliminary data suggest that the SCN may not be necessary for a masking response to light in Nile grass rats, at least under some conditions (*unpublished observations*). Data derived from lesion studies of nocturnal rodents led Redlin (2001) to suggest that the neural pathways controlling masking may be redundant and that multiple retinorecipient brain regions are involved. More recently, Morin (2013b) proposed that ipRGC projections to the SCN may play a key role in regulation of masking as well as photoentrainment. It will be important to determine whether and how this pathway contributes to one or both of these functions in diurnal species.

### Lateral Geniculate Nucleus

The LGN is another region that plays an important role in image and non-image forming visual functions and receives input from ipRGCs in nocturnal (hamsters: Bergstrom et al., 2003; mice: Hattar et al., 2006; Brown et al., 2010; Ecker et al., 2010; rats: Hannibal and Fahrenkrug, 2004) and diurnal (macaque monkeys: Hannibal et al., 2014; Nile grass rats: present study) species. In the Nile grass rat we saw relatively little input from ipRGCs (as indicated by PACAP-immunoreactivity) in either the dLGN or the vLGN (**Figures 6–8**). In this respect, Nile grass rats are very different from nocturnal rodents, in which both the dLGN and vLGN are heavily innervated by ipRGCs (mice: Brown et al., 2010; Ecker et al., 2010; rats: Hannibal and Fahrenkrug, 2004). This may reflect a reduced number of non-M1 ipRGCs in the Nile grass rat retina, as they are the primary ones that project to these regions of the LGN in nocturnal mice (Brown et al., 2010; Ecker et al., 2010). In the most rostral portion of the Nile grass rat dLGN, we did see some PACAP fibers of retinal origin and their distribution resembled that of axons emanating from the M1 cells in mouse retina (Hattar et al., 2006). The presence of these PACAP-ir fibers raises the possibility that ipRGCs play an important role in processing visual information in Nile grass rats, as they appear to do in nocturnal rodents (Brown et al., 2010; Ecker et al., 2010) and in primates and humans (Dacey et al., 2005; Hannibal et al., 2014). These fibers may also modulate masking, as lesions of the dLGN in mice actually enhance masking responses to low intensity light (Edelstein and Mrosovsky, 2001).

In contrast to the dorsal and ventral LGN, the IGL appears to receive dense input from ipRGCs in the Nile grass rat, as it does in other species (mice: Hattar et al., 2006; rats: Hannibal and Fahrenkrug, 2004; hamsters: Bergstrom et al., 2003; macaques (pregeniculate complex): Hannibal et al., 2014). However, some PACAP-containing fibers within the IGL did not originate from the retina. The source of these fibers might be the contralateral IGL or vLGN, as these structures are known to project to the IGL in nocturnal rodents (Morin, 2013a) and have weakly stained PACAP-ir cell bodies (present results). In mice, fibers emanating from M1 and non-M1 ipRGCs are intermixed in the IGL (Ecker et al., 2010). The role of the ipRGC projection to the IGL has not been clearly established, but it is likely to be related to the temporal structuring of daily activity patterns. In nocturnal rodents the IGL is known to be important for communication of both photic and non-photic information to the SCN and it appears to play an important role in modulation of masking through pathways that have not yet been clearly established (Morin, 2013a). In hamsters, IGL lesions enhance negative masking (i.e., the inhibitory effects of light on activity are increased; Redlin et al., 1999), whereas in diurnal Nile grass rats, the positive masking response to light are reversed by IGL lesions (Gall et al., 2013); that is, light reduces locomotor activity in IGL lesioned animals. This indicates that the IGL may contribute to the differences in the masking response of intact diurnal and nocturnal rodents, something likely to be related to ipRGC input to this region.

### Pretectum

Pituitary adenylate cyclase-activating polypeptide fibers of retinal origin were also evident in two areas of the pretectum, the PLi nucleus and olivary pretectal area (OPT; **Figures 8–11**), regions that receive input from ipRGCs in other species as well (Hannibal and Fahrenkrug, 2004; Hattar et al., 2006; Hannibal et al., 2014). The PLi is known in hamsters to both receive input from and send projections to the vLGN, IGL, other areas of the pretectum (including the OPT) and the SC in hamsters (Morin and Blanchard, 1998). The OPT region of the pretectum had a high amount of PACAP-ir fibers coming from the retina in our Nile grass rats (**Figures 8–10**). Interestingly, we only saw these ipRGC fibers in the most rostral portion of the OPT, whereas in nocturnal mice they are present across its full rostrocaudal extent (Hattar et al., 2006). This is particularly interesting because lesions of the pretectum, including the OPT, attenuate the masking response of sleep patterns to light in Norway rats (Miller et al., 1998), and actually reverse masking of activity in diurnal Nile grass rats; that is, light triggers a decrease, rather than an increase, in their general activity (*unpublished data*). Further, effects of light on the OPT are quite different in Nile grass rats and nocturnal mice; specifically, light induces an increase in FOS in the OPT of the former species and a decrease the latter (Shuboni et al., 2015). Thus, it is tempting to speculate that differential responsiveness of the OPT to photic information reaching it through the ipRGCs contribute to differences in masking associated with chronotype.

In nocturnal mice, distinct subtypes of melanopsin cells differentially innervate the OPT. While the shell receives input from M1 cells, the core receives input from non-M1 cells (Hattar et al., 2006; Baver et al., 2008; Ecker et al., 2010). The M1 population can be further divided by which ones express the transcription factor, Brn3b. Specifically, whereas most M1 cells and all non-M1 cells express Brn3b in the adult mouse retina, there are some M1 cells, about 200 (which represents ∼10% of all ipRGCs) that do not (Chen et al., 2011). These cells project extensively to the SCN and moderately to the IGL and are sufficient for both photoentrainment and masking. However, these cells do not project to the OPT and are not sufficient for the PLR, which requires M1 and non-M1 cells expressing Brn3b that project to the OPT (Chen et al., 2011). Whether this is similar in Nile grass rats, needs to be determined.

### Superior Colliculus

In Nile grass rats, as in other species (hamsters: Morin et al., 2003; mice: Hattar et al., 2006; rats: Hannibal and Fahrenkrug, 2004), the SC appears to receive some input from ipRGCs. It is clear that the projection to the SC is crossed, since removal of one eye led to a substantial decrease in PACAP-ir in the contralateral, but not the ipsilateral, SC (**Figure 8**). In mice, both M1 and non-M1 ipRGCs project to the SC (Ecker et al., 2010). The SC plays an important role in directing eye movements in response to visual cues, which suggests that ipRGC projections to this structure may play a role in this aspect of visual processing. It may also contribute to masking, as lesions of the SC increase direct effects of low intensity light on wheel running activity in hamsters (Redlin et al., 2003). Although the SC may not be necessary for masking, it could play an important modulatory role (Redlin et al., 2003). Nothing is currently known about this issue in diurnal species.

## Conclusion

In conclusion, ipRGCs, as defined by the presence of melanopsin, contain PACAP in the diurnal Nile grass rat, as they do in nocturnal rodents (hamsters: Bergstrom et al., 2003; rats: Hannibal and Fahrenkrug, 2004) and diurnal primates (macaque monkeys: Hannibal et al., 2014; humans: Hannibal et al., 2004). Although there were some differences in their distribution across the retina, and in the relative numbers of the different sub-types of ipRGCs, the fundamental features of these cells were the same as those described in other species. The central projections of PACAP-containing cells in the retina to brain regions involved in image and non-image forming visual functions, such as masking, are also very similar across species, including Nile grass rats (**Figure 12**). However, some interesting differences were apparent. For example, the ventral and dorsal LGN appear to receive less input from ipRGCs in Nile grass rats than in nocturnal murid rodents. Furthermore, whereas ipRGCs project to full rostrocaudal extent of the OPT in other species, the caudal OPT does not seem to receive such input in grass rats. Finally, it should be noted that there might be differences within target structures identified here with respect to which cell populations receive direct input from the ipRGCs. Thus, future studies are needed to test the hypothesis that differences in patterns of connectivity between ipRGCs and their targets contribute to differences between diurnal and nocturnal species with respect to their masking responses to light.

## Author Contributions

All authors take responsibly for the accuracy and integrity of the described work. The concept and experimental design were performed by JL, LS, and JH. All authors contributed in some way to the acquisition and analysis of the data. The initial drafting of the work was done by JL and all authors were involved in subsequent revisions of the manuscript.

## Conflict of Interest Statement

The authors declare that the research was conducted in the absence of any commercial or financial relationships that could be construed as a potential conflict of interest.
